# RNAcontext: A New Method for Learning the Sequence and Structure Binding Preferences of RNA-Binding Proteins

**DOI:** 10.1371/journal.pcbi.1000832

**Published:** 2010-07-01

**Authors:** Hilal Kazan, Debashish Ray, Esther T. Chan, Timothy R. Hughes, Quaid Morris

**Affiliations:** 1 Department of Computer Science, University of Toronto, Toronto, Ontario, Canada; 2 Banting and Best Department of Medical Research, University of Toronto, Toronto, Ontario, Canada; 3 Department of Molecular Genetics, University of Toronto, Toronto, Ontario, Canada; 4 Donnelley Centre for Cellular and Biomolecular Research, University of Toronto, Toronto, Ontario, Canada; University of Illinois Urbana-Champaign, United States of America

## Abstract

Metazoan genomes encode hundreds of RNA-binding proteins (RBPs). These proteins regulate post-transcriptional gene expression and have critical roles in numerous cellular processes including mRNA splicing, export, stability and translation. Despite their ubiquity and importance, the binding preferences for most RBPs are not well characterized. *In vitro* and *in vivo* studies, using affinity selection-based approaches, have successfully identified RNA sequence associated with specific RBPs; however, it is difficult to infer RBP sequence and structural preferences without specifically designed motif finding methods. In this study, we introduce a new motif-finding method, RNAcontext, designed to elucidate RBP-specific sequence and structural preferences with greater accuracy than existing approaches. We evaluated RNAcontext on recently published *in vitro* and *in vivo* RNA affinity selected data and demonstrate that RNAcontext identifies known binding preferences for several control proteins including HuR, PTB, and Vts1p and predicts new RNA structure preferences for SF2/ASF, RBM4, FUSIP1 and SLM2. The predicted preferences for SF2/ASF are consistent with its recently reported *in vivo* binding sites. RNAcontext is an accurate and efficient motif finding method ideally suited for using large-scale RNA-binding affinity datasets to determine the relative binding preferences of RBPs for a wide range of RNA sequences and structures.

## Author Summary

Many disease-associated mutations do not change the protein sequence of genes; instead they change the instructions on how a gene's mRNA transcript should be processed. Translating these instructions allows us to better understand the connection between these mutations and disease. RNA-binding proteins (RBP) perform this translation by recognizing particular “phrases” that occupy short regions of the transcript. Recognition occurs by the binding of the RBP to the phrase. The set of phrases bound by a particular RBP is defined by the RNA base content of the binding site as well as the 3D configuration of these bases. Because it is impossible to assess RBP binding to every possible phrase, we have developed a mathematical model called RNAcontext that can be trained by measuring RBP binding strength on one set of phrases. Once trained, this model can then be used to accurately predict binding strength to any possible phrase. Compared to previously described methods, RNAcontext learns a more precise description of the 3D shapes of binding sites. This precision translates into more accurate generalization of RBP binding preferences to new phrases and allows us to make new discoveries about the binding preferences of well-studied RBPs.

## Introduction

RBPs act in the post-transcriptional regulation (PTR) of gene expression by binding to target RNAs to control splicing, stability, localization and translation. Recent draft networks of RBP-transcript physical interaction in yeast [1], fruit flies [2], and humans [3] reveal a complex and combinatorial pattern of RBP targeting and supports an RNA regulon model [4] in which *cis*-regulatory transcript sequence dictates the post-transcriptional fate of an mRNA at multiple, distinct stages of regulation. Deciphering this operon code as well as the role of individual RBPs in post-transcriptional regulation requires the detailed characterization of the binding preferences of RBPs.

We have recently introduced the *RNAcompete* assay [5], a microarray-based *in vitro* method to estimate the binding affinity of selected RBPs to a defined population of short RNA sequences. RNAcompete, along with *in vivo* methods such as RIP-seq [6] and CLIP-seq [7], can be used to determine binding preferences of individual RBPs for a large number of RNA sequences. Motif representation generated from these data can be used to scan mRNA transcripts to identify potential RBP binding sites. However, this step can prove challenging because many RBPs show a preference for both specific sequences and secondary structure contexts in their binding sites [8]–[12].

Despite these structural preferences, motif finding algorithms that ignore RNA secondary structure work surprisingly well for some RBPs. This approach has been successful for both *in vitro* and *in vivo* binding data [1], [2], [5], [13], [14]. For example, structure-naive motif finding applied to mRNAs targeted by yeast proteins Puf3p and Puf4p recover sequence preferences confirmed by crystal structures of the RBP-RNA complexes [15], [16]; and motif models for YB-1, SF2 and PTB fit to *in vitro* binding data from the RNAcompete assay predict their *in vivo* targets with high accuracy [5].

However, this approach can give misleading results when an RBP has non-trivial structural preferences. For example, Vts1p is a yeast RBP that preferentially binds 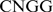 loop sequences within RNA hairpins [17], however, this binding preference can be difficult to detect without consideration of this structural preference (e.g., [1]). RBP motif finding can made more reliable by training structure-naive algorithms only on RNA sequence likely to be in the preferred context [9], [18]. For example, Foat and Stormo [18] could reliably extract the Vts1p sequence binding preferences from *in vivo* binding data by using only loop sequences (from likely hairpin loops) to train the MatrixREDUCE[19] motif finding algorithm. Similarly, the MEMERIS [9] algorithm adapts the MEME [20] motif finding algorithm to search for RNA motifs enriched in single-stranded regions by assessing a prior on each word according to its structural accessibility. MEMERIS predicts binding sites more accurately than MEME for a number of proteins, including the mammalian stem-loop binding RBP U1A. However, applying this strategy only allows a single, pre-defined structural preference to be queried. Ideally, an RBP motif finding method should consider multiple possible structural contexts simultaneously, and detect the relative preferences of a particular RBP for each.

Covariance models (CMs) [21] are RNA motif models often used for modeling families of ncRNAs (e.g., [22]) and have the capacity, in theory, to represent both the sequence and (arbitrary) structure preferences of RBPs. However, CMs have a reported tendency to overpredict secondary structure [23]. Indeed, recent CM-based motif models of Puf3p, Puf4p, and HuR [24], [25] predict they preferentially bind RNA hairpins and contradict structural, *in vitro* and *in vivo* evidence [5], [12], [26], [27], that they bind unstructured ssRNA.

We present a new strategy for modeling RBP binding sites that learns both the sequence and structure binding preferences of an RBPs. Our method assumes that the primary role of RNA secondary structure in RBP binding is to establish a structural context (e.g., loop or unstructured) for the RNA sequence recognized by the RBP. As such, we annotate each nucleotide in terms of its secondary structure context (e.g., paired, in a hairpin loop or bulge). Cognizant of the fact that a given RNA sequence can have multiple, distinct stable secondary structures, this annotation takes the form of a distribution over all its possible contexts. These distributions are estimated using computational models of RNA folding. Our new model can be discriminatively trained (as [19], [28], [29]) thus facilitating its use with either binding affinity data or sets of bound sequences.

We apply RNAcontext to several RNA-binding affinity datasets, demonstrating that it can infer the RBP structure and sequence-binding preferences with greater accuracy than other motif-finding methods. RNAcontext recovers previously reported sequence and structure binding preferences for well-charactered RBPs including Vts1p, HuR, and PTB and predicts new structure binding preferences for FUSIP1, SF2/ASF, SLM2, and RBM4.

## Methods

We now present our approach for discovering RNA sequence and structure binding preferences of RBPs. This section is organized as follows: we first describe how we annotate an RNA sequence in terms of its structural context. Then, we discuss the details and the mathematical formulation of our motif model. Lastly, we describe our procedure for fitting the RNAcontext motif model. Because part of our model is derived from prior work on DNA motif finding, we summarize this work in Protocol S1. Source code in C++ for RNAcontext is available online at http://morrislab.med.utoronto.ca/software.

### Structural annotation of RNA sequences

We use computational algorithms to predict RNA secondary structures though our algorithm can use experimentally determined RNA secondary structures when they are available. Instead of focusing on the single minimum free energy structure which is often not representative of the full ensemble of possible structures [30], we consider the ensemble of secondary structures that the RNA can form.

In the experiments reported here, we used SFOLD [30] to estimate the marginal distribution at each nucleotide over structural contexts (e.g. paired, unpaired, hairpin loop) for each position of the sequence by sampling a large number of structures for the sequence according to the Boltzmann distribution. We annotated each base in each structure using our context annotation alphabet (described below) and then we set the structural context distribution (hereafter called the annotation profile) to be the empirical annotation frequencies for that base across these samples. In all experiments described herein we used 1,000 samples.

Our motif model can use any annotation alphabet. However, in this manuscript, we only use the alphabet P, L, U, M indicating that the nucleotide is paired (P), in a hairpin loop (L), or in an unstructured (or external) region (U). The last annotation, M, stands for miscellaneous because we combine the remaining unpaired contexts (i.e., the nucleotide is in a bulge, internal loop or multiloop). This group of structural contexts are expressive enough to distinguish most known RBP structure preferences.

### Motif model

**Figure 1:**
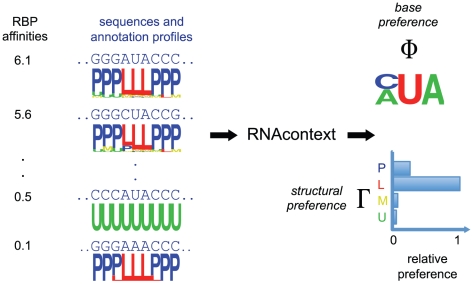
Overview of RNAcontext method: the input, parameters and motif model. The input to RNAcontext consists of a set of sequences together with their associated structure annotation profiles (estimated using SFOLD) and RNA-binding affinity estimates for the given RBP. The motif model has sequence parameters (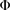) and structure parameters (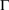) where the former describes the inferred base preferences (as a PWM) and the latter describes the relative structural preferences of the RBP to different structural contexts. Shown is a toy example, where the sequences with highest binding affinities have *AUA* or *CUA* in hairpin loop context and the sequences with lowest binding affinities either lack the sequence motif or contain the sequence motif in another structural context. By learning a motif model that predicts the input affinities, RNAcontext would infer the sequence and RNA structure preferences as shown on the right part of the figure.

Figure 1 shows an overview of our method. A set of sequences together with SFOLD predicted structure annotation profiles serve as input to the model. Each input RNA molecule is scored using the sequence and structure parameters. Formally, let 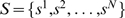 represent the input set of sequences and let 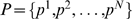 be a set of real-valued matrices that represent the annotation profiles of the corresponding sequences. We use A to represent the alphabet which is composed of the structure features and associate each annotation in A with one of the rows of 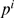. The columns of 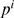 correspond to the positions in sequence 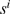 and are discrete probability distributions over the annotations in the alphabet A.

Let 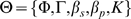 represent the model parameters where 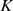 is the width of the binding site, 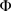 is a position weight matrix (PWM) of sequence features with dimensions 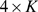, 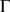 is a vector of structure annotation parameters with one element for each letter in the alphabet 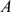. For instance if 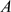 = 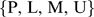 then 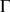 will consist of parameters (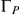, 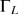, 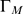, 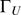) for the structure annotations 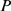, 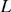, 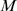 and 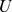, respectively. Lastly, 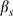 and 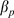 stands for the bias terms in sequence affinity model and structural context model respectively.

We use 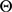 to assign a score, 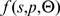, to a sequence 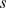 and its corresponding annotation profile 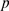. For an RBP with a binding site of width 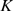, following [31], we define 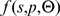 as the probability that at least one of its subsequences of length 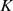 (which we call 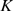-mers) is bound by the RBP, that is:
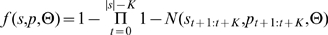
(1)where 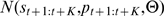 is an estimate of the probability that the 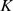-mer with base content 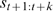 and with structural context defined by the probability profile matrix 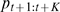 is bound. Here, 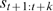 indicates the subsequence of 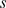 between 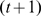-th element and 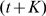-th element, inclusive, and 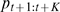 is a matrix whose columns are the annotation distributions for each of the bases between 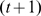-th and 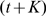-th position. We set 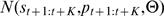 to be the product between a term that depends only its base content, 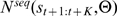, and one that depends only upon its structural context 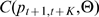, i.e.:
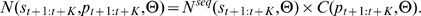
(2)We interpret the term 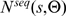 as an estimate of the probability that the RBP will bind 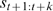 in the ideal structural context. We use a standard biophysical model [28], [31], [32] to define 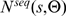 (please see Protocol S1 for more details on this model):
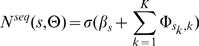
(3)where 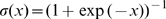 is the well-known logistic function. The logistic function takes value 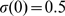 at 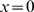 where it is an approximately linear function of 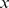, but it quickly saturates toward 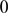 for negative 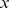 and 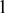 for positive 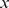. We also model the structural context term using a logistic function of the sum of the structure parameters weighted by corresponding profile values plus a bias term 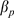:
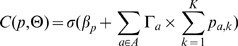
(4)where 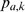 represents the probability that the base at position 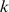 of 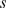 has structural annotation 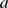. In a preferred structural context, as represented by an annotation 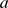 associated with large positive values of 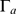, the score 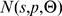 for a 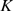-mer 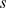 approximately equals 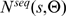 and is thus determined by the base content 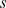. Whereas in a highly disfavored structural context, as represented by highly negative values of 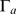, 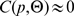 and therefore the score 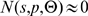 regardless of 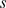 because 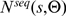 is bounded above by 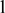 for all 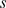. So, the context term licenses binding in favored structured contexts.

In the following section, we describe how to estimate the parameters of our motif model from binding data. However, in theory, our motif model has the flexibility to represent many different modes of RBP binding. For example, the binding preferences of RBPs, like HuR and Vts1p, that bind their preferred sequences within a specific structural context, unstructured (U) [33] and hairpin (H) [17] respectively, can be represented by setting 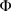 to match their sequence binding preferences and 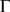 to have negative elements except for the elements of 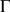 that corresponds to their preferred structural context (either 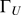 or 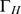 respectively). The binding preferences of RBPs, like U1A, that have multiple preferred contexts (e.g., hairpin loops [34] or unstructured ssRNA [35]) can be captured by setting 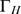 and 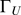 to large positive values. RBPs, like Staufen, that bind dsRNA without obvious sequence preferences [36], can be represented by setting the elements of 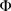 to constant values, and setting 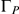 to a large positive value. Similarly, RBPs without strong structure preferences can be represented by setting the elements of 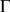 to zero and setting 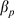 to a large positive value. Our model thus extends previous efforts that model RBP binding preferences [8] by associating each RBP with a single preferred structured context which is required for binding.

In the next section, we describe how we can estimate the sequence and structure preferences of new RBPs by training our model using RBP binding or RBP binding affinity data for short RNA sequences.

### Parameter estimation

We learn 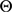 by using our model to attempt to reproduce the observed affinity data 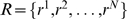 given the associated sequences 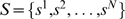. In particular, we model the affinity 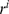 of a sequence 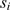 as a linear function of the sequence score 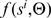 with unknown slope 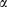 and 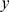-intercept 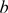 and search for settings of 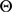, 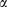, and 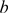 that minimize the sum of the squared differences between the measured affinity 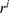 and our predicted affinities 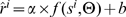. When we only know whether or not a given sequence is bound we use 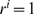 for all bound sequences and 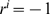 for sequences not bound. This formulation leads to the following least squares cost function, 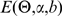, that we attempt to minimize with respect to 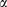, 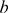, and 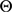 using the L-BFGS method [37]:
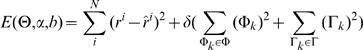
(5)Here, we have added a regularization term scaled by a small constant 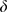 to avoid indeterminancy thus ensuring a unique global minimum. We use the same value of this constant in all experiments. We use the bound constraints feature of the L-BFGS-B package to constrain 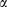 to take positive values so that the estimated affinity increases as a function of the sequence score.

The cost function optimized by RNAcontext is multimodal, so different initializations can generate different results. For the experiments reported here, we used ten different initialization for each motif width. For motif lengths, 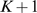, longer than the minimum length, two of these initial settings are generated by taking the optimal 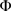 matrix learned for 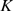 and adding a column of zeros to its left and right sides, respectively. The elements of 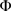 matrix for the other initializations are randomly sampled uniformly between −0.05 and 0.05. In all cases, the other parameters (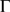, 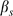, 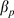, 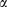, 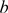) are randomly sampled uniformly between −0.05 and 0.05.

## Results

### Dataset

We evaluated our motif model on *RNAcompete*-derived datasets [5] comprised of the measured binding preferences of nine RBPs (i.e., HuR, Vts1p, PTB, FUSIP1, U1A, SF2/ASF, SLM2, RBM4 and YB1) to a pool of 213,130 unique short (29- to 38-nt) RNA sequences (see GEO record GSE15769 and/or Agilent array design: AMADID # 022053 for the array design and data). *RNAcompete* estimates an RBP's binding affinity for each sequence in an RNA pool based on the relative enrichment of that RNA sequence in the bound fraction versus the total RNA pool (as measured by transformed microarray intensity ratios).

The RNA pool can be divided into two separate sets, Set A and Set B, that each individually satisfy the following constraints: (i) each loop of length 3 to 7 (inclusive) is represented on at least one sequence flanked by RNA stems of 10 bases; and (ii) a population of “weakly structured RNAs” wherein each possible 7-mer is represented in at least 64 different sequences that have high folding free energy, and therefore are linear or form weak secondary structures. We call the group satisfying the first constraint the *stem-loop* sequences. This group also contains 60% of the possible length eight loops. We call the sequences satisfying the second constraint the *weakly structured* sequences. There is no overlap between the stem-loop and weakly structured sequences.

So in summary, there are two different groups of stem-loops, one in Set A and one in Set B, and similarly, two different groups of weakly structured sequences. It is important to note two things. First, though we attempted to design these sequences to be linear or hairpins, there are many unintended structures represented in the pool. For example, some of the sequences contain bulge or internal loops and some of the weakly structured sequences contain stem-loops. Second, no two sequences within the pool share a common subsequence more than 12 nt long. The design and properties of these sequences are described in greater detail in [5].

The division of the RNA sequence pool into Set A and Set B provides a natural strategy for evaluating our motif models using two-fold cross-validation: we train our algorithm on one of the two sets and test its predictive power on the other set. This strategy provides us with two independent measurements of performance on non-overlapping training sets. Table S1 contains more information on the sizes and compositions of the sequences used for training and testing. The categorizations “Positive”, “Negative”, and “Other” that appear in this table are described below. Note due to stringent RNAcompete quality controls, some affinity data is missing for some of the sequences, so the numbers in the table do not add up to 213,130 for each RBP.

### Justification of choice of motif models for comparison

We evaluated RNAcontext against two other motif finding methods: MEMERIS [9] and MatrixREDUCE [19]. MEMERIS and RNAcontext use similar approaches to model the structural context of an RNA binding site except that MEMERIS only models a single structural context where RNAcontext considers multiple contexts simultaneously. In contrast, MatrixREDUCE does not consider the structural context of RBP binding sites and therefore can help determine the value of considering structural context in RNA motif finding. Additionally, MatrixREDUCE outperforms many standard DNA motif finding algorithms on a similar experimental assay [38] and therefore provides a strong algorithm to benchmark to compare RNAcontext and MEMERIS against. Also, like RNAcontext, MatrixREDUCE learns its motif model by trying to predict RNA sequence affinity whereas MEMERIS searches for motif models enriched in a set of bound sequences.

### Fitting motif models

In this subsection we describe our protocol for using the training data to fit the MEMERIS, MatrixREDUCE and RNAcontext motif models. Note that for all three methods, we fit all parameters, including those of the motif models and any free parameters (like motif width), using the training data. One of the free parameters that we consider for each method is whether it is better to train their motif model on the whole training set, or a defined subset of the training set. All of the free parameters that we consider for each method are described below. For every setting of the free parameters, we fit one motif model. The “best” motif model for each method was selected based on its ability to correctly classify “Positive” and “Negative” RNA sequences in the training set, as defined in the next paragraph. The final result of training is a single motif model for each method that we then evaluate on the test set.

The parameters of some motif models are fit using subsets of the training set because: (i) MatrixREDUCE does not model RNA secondary structure and it is possible that its performance would degrade when trained on stem-loop sequences (most of whose bases are paired); and (ii) MEMERIS takes as input a set of “bound” sequences that contain RBP binding sites. For MEMERIS, “bound” sequences are selected using a manual cutoff that captures the right tail of the distribution of the RNAcompete affinity estimates. We used a different cutoff for each RBP and each training set and the number of bound sequences ranged between 234 and 792 for the RBPs analyzed. Additionally, we used these bound sequence as the “Positive” sequences for Area Under the Precision-Recall Curve (AUC-PR). For the “Negative” sequences required by the AUC-PR calculation, we used those with estimated affinities below the median affinity of the training set. Any sequence not deemed a “Positive” or “Negative” is labeled as “Other” in Table S1. We score each motif model's performance by using it to estimate RNA-binding affinities for the “Positive” and “Negative” sequences and then evaluating classification accuracy using the AUC-PR. Because each algorithm models RBP binding preferences in a slightly different manner, in this section, we also describe how we estimate RNA-binding affinity for each sequence using the motif models for each algorithm.

For each method, we trained two sets of motif models. One set of models was fit using the full training set which consists of all RNA sequences in the training set for MatrixREDUCE and RNAcontext and all bound RNA sequences in the training set for MEMERIS. The other set of models was fit using only the weakly structured sequences in the training set (i.e., removing the stem-loops).

We consider a wide range of combinations of free parameters for MEMERIS. In particular, we tried all possible combinations of the following free parameter choices: the EF and PU options for measurement of single-strandedness; OOPS, ZOOPS and TCM options for the expected number of motifs per sequence (see Protocol S1 for details on these options); motif lengths between 4 and 12 nts (inclusive); different values for the pseudocount parameter (i.e. 0.1, 1 and 3); and selecting the training set using a permissive cutoff (i.e., the bound sequences) or a stringent cutoff (i.e., the top half of bound sequences). The final option means that we consider four different subsets of the training set for each setting of the other free parameters (i.e. permissive/full, stringent/full, permissive/weak, stringent/weak). In total, we fit 648 different motif models for MEMERIS for each training set. We estimate affinity for each RNA sequence using a MEMERIS Position Frequency Matrix (PFM) motif model by following an approach similar to that used by MotifRegressor [39]. Namely, we calculated the foreground probability of a K-mer under the product-multinomial distribution defined by the PFM and calculated the background probability using a third-order Markov model trained on either the full training set (or test set, as appropriate). As explained in Protocol S1, the ratio of the foreground and background probabilities is an estimate of the relative affinity of the RBP for that K-mer. For some RBPs, when it led to a performance increase, we also multiplied this affinity by the probability that the site was accessible, as determined using the optimized settings of the EF/PU and pseudocount parameters for that training set. To estimate the affinity of the entire sequence, we summed its k-mer relative affinities. Note that we also tried MAST [40] to score the sequences using MEMERIS's motif models but test set performance decreased (data not shown).

We used MatrixREDUCE to generate single motifs with widths ranging from 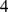 to 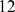 by setting 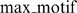 to 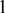. The MatrixREDUCE program automatically selects the appropriate motif width, so we only needed to choose between two different MatrixREDUCE motifs on each training set (one trained on the full set and the other only on the weakly structured sequences). Note that MatrixREDUCE's PSAM motif model directly estimates relative binding affinity of the RBP for each k-mer, so to estimate RNA sequence affinity, we summed PSAM scores for each constituent k-mer.

We ran RNAcontext with motifs width ranging from 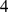 to 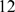, thus creating 18 motif models per training set, and used equation (1) to score RNA sequences using these models.

For all three methods, for each training set, we used the AUC-PR on training set “Positives” and “Negatives”, to select the best single model among the fitted models. The free parameters settings for the selected models are in Table S2.

### Performance evaluation

RNAcontext achieved higher average AUC-PR values than MEMERIS and MatrixREDUCE on all of the nine RBPs analyzed (Table 1). It also had significantly higher AUC-PRs than either method on 15 of the 18 test sets encompassing seven of the nine RBPs (the largest P-value was 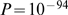, Wilcoxon's sign-rank test on the AUC-PR values of 1,000 bootstrap samples; See Table S3 for the complete results of bootstrap analysis).

**Table 1:** Comparison of predictive accuracy of three motif finding models using both weakly structured and stem-loop sequences in the test set.

Proteins	RNAcontext	MEMERIS	MatrixREDUCE
RBM4	**0.91**	0.43	0.63
FUSIP1	**0.53**	0.31	0.32
Vts1p	**0.65**	0.58	0.56
YB1	**0.17**	0.07	0.11
SLM2	**0.81**	0.49	0.77
SF2	**0.70**	0.50	0.66
U1A	**0.30**	0.27	0.21
HuR	**0.96**	0.74	0.94
PTB	**0.69**	0.26	0.67

The values show the average AUC-PR across two test sets and bold values indicate the best performing method. Rows are sorted by decreasing relative gain of RNAcontext to the best of MEMERIS & MatrixREDUCE. For all the methods, displayed values were calculated using the single best motif model for each method chosen based on the two training set performance. According to the Wilcoxon's sign rank test performed on paired AUC-PR values across 1,000 bootstrap samples from the test set results of the three methods, all differences between RNAcontext AUC-PR and that of the other algorithms are statistically significant (the largest P-value is 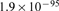, see Table S3 for the complete results of bootstrap analysis) except for the differences on PTB and U1A.

The improvement in AUC-PR of RNAcontext compared with MatrixREDUCE is largest for proteins whose preferred structural context is less common in the RNA pool, reflecting the fact these are the hardest binding sites for MatrixREDUCE to predict. For example, RNAcontext performs much better than MatrixREDUCE on Vts1p which binds to CNGG in the loop of an RNA stem-loop. This sequence appears frequently outside of a loop context in the RNA pool. We also see large improvements for RBM4 that binds to CG containing sequences in an unpaired context, likely because these sequences often appear in stems. In contrast, HuR's binding site is U-rich and, as such, is rarely paired in either the training or test set. In this circumstance, MatrixREDUCE's lack of a structural model does little harm to its performance.

Although MEMERIS has higher average AUC-PR than MatrixREDUCE for stem-loop binding proteins Vts1p and U1A, reflecting the value of its model of structural context, its average AUC-PR was otherwise worse than that of MatrixREDUCE and always worse than that of RNAcontext. This is likely due to its inability to make use of the affinity data associated with each sequence. One consequence of this is that it can only trained on a small subset of the data. Some of the loss in AUC-PR on the test set may also be due to overfitting because of the large number of parameter combinations that needed to be considered.

### The predictive value of structural context

**Figure 2:**
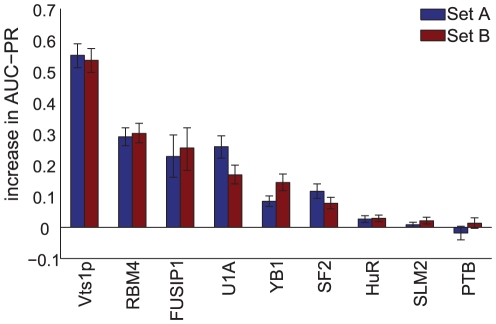
Change in predictive accuracy of RNAcontext due to the representation of RNA structural context. Bar graph shows the increase in AUC-PR of RNAcontext with 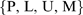 alphabet compared with the model without RNA structural context for each of nine RBPs using the two test sets. Error bars show 95% confidence interval of the difference estimated from 1,000 bootstrap samples of the test set.

Having established that RNAcontext can capture RBP binding preferences better than comparable motif models that either do not model RNA secondary structure (MatrixREDUCE), or use a limited representation (MEMERIS), we then attempted to confirm that the added predictive value was due to the incorporation of structural context, rather than differences in how we estimate sequence affinity. To do this, we compared our model based on the 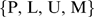 structural annotation alphabet to a simplified version of our model whose alphabet only contains a single letter (i.e. all bases have an identical structural annotation). As in previous sections, the two models were fit to the data for each of the nine RBPs using a variety of motif widths (4–12). Also, as before, we used training set AUC-PR to choose the optimal motif width and to choose between the full training set and only the weakly structured sequences. After selecting the single best model for the two methods, we compared RNAcontext against the structure-naive model using AUC-PR on the full test set. To assess the significance of difference in AUC-PR, we used 95% confidence interval of the difference estimated from 1,000 bootstrap samples. Figure 2 shows these differences for nine RBPs on the two cross-validation test sets. Using structural context lead to a significant improvement in AUC-PR for eight of the nine RBPs. In some cases, the difference was dramatic, particularly for Vts1p, RBM4, FUSIP1 and U1A.

### Position-specific scoring matrices provide good approximations of sequence binding preferences for six RBPs

**Figure 3:**
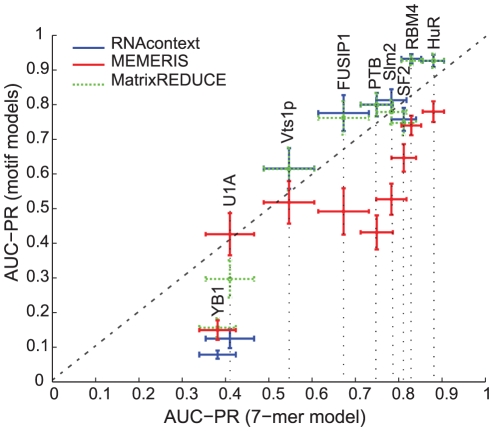
The predictive value of the fully-specified 7-mer model (i.e., the *RNAcompete* model) with respect to the motif models. This scatter plot compares the performance of the *RNAcompete* model to other motif models where the training and test sets are formed using only weakly structured sequences. The x-axis shows the AUC-PRs of *RNAcompete* and the y-axis shows the AUC-PRs of the three motif models: RNAcontext (blue), MEMERIS (red) and MatrixREDUCE (green). Each point corresponds to the mean AUC-PR of 1,000 bootstrap samples, averaged across the two test sets. The error bars indicate the 95% bootstrap confidence interval.

We then sought to assess the accuracy of position-specific scoring matrix (PSSM) approximations of RNA-sequence binding preferences by comparing the predictive power of inferred 7-mers affinities to that of the three PSSM-based models. We trained a “fully-specified 7-mer model” that estimates the binding affinity of an RBP for every 7-mer by taking a trimmed average of the transformed intensity ratios of the weakly-structured sequences that contain the 7-mer in the training set (see [5] for more details of this model). We then used these estimated affinities to assign a score to RNA sequences longer than seven nucleotides, by taking the mean of the affinities of each 7-mer in each sequence in the test set. We also trained and evaluated RNAcompete, MatrixREDUCE and MEMERIS motif models as previously described except that we always restricted the training and test sets to the weakly-structured sequences. We used only the weakly-structured sequences in this comparison so that we could more readily evaluate the ability of PSSM models to assess sequence binding preferences separately from each method's ability to capture RBP structure binding preferences. Figure 3 compares the 7-mer model against the three methods with respect to average AUC-PR on the test sets. PSSM-based motif models perform significantly better than the 7-mer model for every RBP except U1A (and only on test set A), YB1, and SF2/ASF (the Wilcoxon sign-rank P-values for the best PSSM motif model are all less than 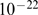). Notice that because MatrixREDUCE performs significantly better than the RNAcompete method for five of the nine RBPs, this performance gain can not be explained by the incorporation of structural context in RNAcontext.

### The sequence and structure binding preferences for seven RBPs

**Figure 4:**
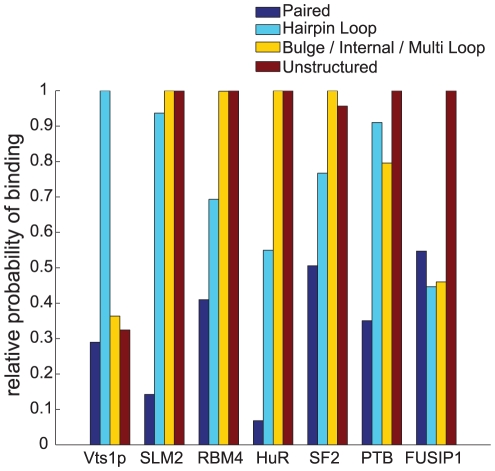
Relative RNA structure preferences inferred by RNAcontext. Y-axis indicates the ratio between the context scale factor 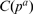 (see equation 4) for a structural context with probability one for the indicated annotation (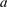) for all bases (i.e. 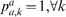) to the context scale factor 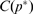 for the best possible structural context for the RBP (i.e. 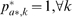 where 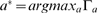). Displayed are ratios across parameters learned from the training set containing all the sequences (i.e. both Set A and Set B). For Vts1p, the most preferred context was predicted to be hairpin loop and this is consistent with the known binding preferences. SLM2, RBM4, and HuR have similar preferences, and predicted to bind regions that are not paired.

**Figure 5:**
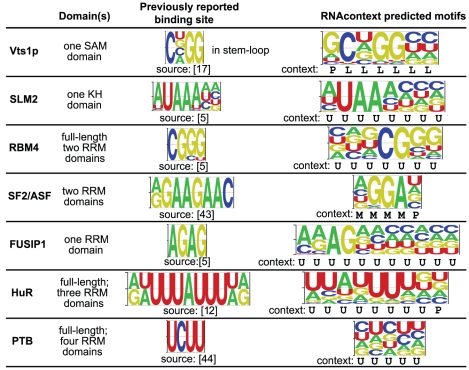
Inferred sequence and structure binding preferences for seven RBPs. RNA-binding domains of the proteins are displayed on the second column and previously reported binding sites are displayed on the third column for reference. RNAcontext predicted sequence parameters are shown as a PFM (fourth column). Also, an estimate of the preferred structural context for each base is displayed underneath each of the logos.

Having established that RNAcontext accurately predicts the *in vitro* affinity for seven of the nine RBPs (with the exception of YB-1 and U1A), we applied RNAcontext to the entire dataset to make the best possible prediction for their binding preferences. The results are shown in Figure 4 and Figure 5. Figure 4 shows the relative structural context preference of each RBP. RNAcontext's predicted structural preferences are consistent with co-crystal structures for Vts1p [17] (loop) and PTB [41] (ssRNA) and *in vitro* and *in vivo* binding data for HuR [5], [8], [12]. RNAcontext also predicts new structural preferences for SLM2, RBM4 and SF2/ASF. Of particular interest, is that RNAcontext predicts that SF2/ASF has a slight preference for RNA binding sites in bulges, internal loops, and/or multiloops (the M annotation). For FUSIP1, we report the motif model trained using only the weakly structured sequences even though the model trained on the full set (shown in Figure S1) had higher AUC-PR. As mentioned in the legend of Figure S1, we could not rule out the possibility that this model reflected an artifact of our pool design despite the fact that the two models both suggest that FUSIP1 prefers its binding site to be 5′ to an RNA stem.

Figure 5 compares the motif logo representations (generated by Enologos software[42]) of RNAcontext's 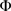 parameters with previously reported motifs for those RBPs. To derive the energy parameters required by Enologos, we uniformly rescaled the elements of the 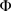 matrix so that 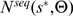 of the optimal binding site, 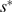, would be 0.5 (as suggested by [31]). Underneath each of the logos for the RNAcontext motifs, we have displayed an estimate of the preferred structural context for each base. In order to identify this context, we found the top 20 best scoring k-mers in the test set under each motif model, averaged the annotation profiles for these 20 k-mers and deemed the annotation with the highest average frequency to be the preferred context for each position in the k-mer. These estimates recover the fact that the Vts1p binding site (CNGG) occurs at the 5′ end of the hairpin loop. Our RNAcontext motifs match previously reported binding sites [12], [17], [43]–[45] and the motifs that we have previously derived from the *RNAcompete* data[5].

### *In vivo* confirmation of RNAcontext motif for SF2/ASF

In both Figure 4 and Figure 5, we observe a preference for the M structural context for the SF2/ASF motif. This preference has not been previously reported for SF2/ASF [43]. To confirm this unusual preference, we collected data on the *in vivo* targets of SF2/ASF from [13]. These targets were generated using the CLIP-Seq assay and consist of 296 short RNA fragments that cross-link to the protein in cultured cells which we call “bound”; and 314 transcript sequences not observed to cross-link which we call “unbound”. These data supported our inferred structure preferences for SF2/ASF. In particular, by manual inspection, we discovered a number of cases of the RNAcontext motif within bulge and internal loops within the bound sequences. Also, using our model trained on the RNAcompete data, we were able to distinguish between bound and unbound sequences with higher accuracy using our model (AUC-PR 0.915) compared with the version of our model with a single letter annotation alphabet (AUC-PR 0.898) and MatrixREDUCE (AUC-PR 0.898). Furthermore, when we train our RNAcontext model on the *in vivo* data, assigning bound sequences an affinity of 1 and unbound ones an affinity of −1, we recover the same structural preference for SF2/ASF (Figure S2).

## Discussion

We have demonstrated that RNAcontext represents an advance over existing methods for modeling mRNA-binding protein binding preferences. Motifs learned by RNAcontext more accurately predicted a held out *in vitro* binding dataset for all of the nine RBPs tested. Seven of these differences were statistically significant. As expected, the size of an improvement depends on the relative representation of the preferred binding site in the preferred structural context (or contexts) in the RNAcontext dataset.

RNAcontext motif models reflect previously reported sequence and structure preferences for well-studied RBPs like HuR, Vts1p and PTB and predict new structure binding preferences for SLM2, RBM4 and SF2/ASF. RNAcontext's predictions are supported by *in vivo* binding data for SF2/ASF: the RNAcontext *in vitro* motif model more accurately predicts *in vivo* binding of SF2/ASF, and RNAcontext motif models trained using the *in vivo* data recover the same structural context preference. We expect similar success with our other new predictions because, as we have previously established (in [5]), binding preferences inferred from RNAcompete data are consistent with *in vivo* binding preferences and that more accurate prediction of RNAcompete-measured binding affinity translates into more accurate prediction of *in vivo* binding.

We have also provided evidence that the position-specific scoring matrix (PSSM) motif representation is a better approximation for the RNA binding preferences of RBPs than it is for dsDNA binding preferences of TFs. In particular, in previous work [38], using a similar evaluation framework, we had found that that a “fully-specified 8-mer model” trained on protein-binding microarray (PBM) [46] data had greater predictive power for 7 of 10 TFs than a set of standard DNA motif-finding algorithms, including MatrixREDUCE, trained on the same data. These observations were consistent with many others (e.g., [47]–[49]) that PSSMs were inaccurate approximations dsDNA binding affinities for the majority of TFs. In Figure 3, we show that the opposite holds for RNA-binding data: PSSM models learned by MatrixREDUCE, which does not consider RNA structure, had greater predictive power than a fully-specified 7-mer model for a majority of RBPs. Although the sample size is small, this result may reflect the increased flexibility of RNA compared with dsDNA which may permit more independent movement and recognition of individual bases. Our observations further suggests that modifications of the basic PSSM model made for TFs that incorporate interactions between bases may not be as indispensible for modeling RBP binding preferences. Note that our conclusions here differ from our previous analyses on the same data [5]. We suspect that this difference is due our use, in the present study, of motif finding methods that take full advantage of the affinity data associated with each sequence. Indeed, MEMERIS, one of the algorithms we also used in [5] performed worse than the fully 7-mer model in Figure 3 for eight of the nine RBPs.

In summary, we have introduced a new motif model of RBP binding preferences and a corresponding algorithm for fitting this model to quantitative estimates of RBP binding affinity for short RNA sequences. Our RNAcontext model makes use of a new technique for representing RNA structure based on a structural context alphabet that we use to annotate individual bases of RNA sequence. This representation is particularly amenable to modeling RBP binding preferences. Although we provide a pipeline to annotate RNA sequences according to the PLUM alphabet, our motif finding code does not require a particular structural context annotation alphabet for bases or even a particular RNA structure prediction method. Hence, RNAcontext can easily be expanded to integrate more parsimonious annotations of structural context or improvements in RNA structure prediction methods.

## Supporting Information

Protocol S1 A brief review of DNA motif finding.
(0.16 MB PDF)

Figure S1 Inferred sequence and RNA structure binding preferences for FUSIP1 using all the sequences as the training set. A) Predicted sequence parameters are shown using a sequence logo representation. An estimate of the preferred structural context for each base is displayed underneath the logo. B) The bar graph shows the relative RNA structure preferences of FUSIP1. Note that all sequences in the RNA pool, including the stem-loop sequences, have an unpaired 5′-AGA or 5′-AGG (the initiation sequence for T7 promoter) at their 5′ end. In all stem-loop sequences, this initiation sequence is followed by a G because the bottom base pair of every stem-loop is G-C. Since AG(A/G)G is very similar to previously reported FUSIP1 binding sites [5], [45], we were concerned that this artifact of the pool design had an impact on the model of FUSIP1 binding preferences fit to the full training set. However, even in the model fit only to the weakly-structured sequences shown in Figure 4, there is a slight preference for the paired context compared to L and M.
(1.04 MB EPS)

Figure S2 Inferred sequence and RNA structure binding preferences for SF2. RNAcontext is used to infer binding preferences of SF2 from in vivo data [13] A) Predicted sequence parameters are shown use a sequence logo representation and an estimate of the preferred structural context for each base is displayed underneath the logo. B) The bar graph shows the relative RNA structure preferences of SF2.
(0.90 MB EPS)

Table S1 Properties of the sequences in the input sets. The composition of Sets A and B in terms of relative proportions of stem-loops and weakly structured sequences among their Positive, Negative and Other groups. The input sets are partitioned into these three groups according to their RNAcompete-measured affinities. The sequences with affinities above a threshold are defined as Positive; the sequences with affinities below the median affinities over all the sequences in the given set are defined as Negative and the remaining sequences are placed in the Other group. Within each group, the number of weakly structured sequences and stem-loops are displayed. For RNAcontext and MatrixREDUCE all the sequences in Positive, Negative and Other categories are used for training whereas when running MEMERIS, only Positive sequences are used for training. The test sets are comprised of all sequences in the Positive and Negative groups.
(0.01 MB PDF)

Table S2 Details about the chosen models for RNAcontext, MEMERIS and MatrixREDUCE. Optimal free parameter settings for RNAcontext, MEMERIS and MatrixREDUCE. The column Set describes the training set and contains either weak or full where weak indicates that motifs were trained on the weakly structured sequences and full indicates that motifs were trained on the full set of sequences. The columns, MW-A and MW-B, show the selected motif length for the test sets A and B respectively. There is an extra other column for MEMERIS which shows the other free parameters that are chosen. Namely, EF and PU are two different ways to measure single-strandedness of a region; OOPS (exactly one motif occurrence per sequence), ZOOPS (zero or one motif occurrence per sequence), and TCM (zero or more motif occurrence per sequence) are options (-mod) that indicate the expected number of motifs per sequence. The values in the next column (i.e. 0.1 or 1) are the chosen pseudocount parameters among the available values 0.1, 1, 3. The lower the pseudocount value, the more impact the single-strandedness of the binding site has in the model. Two different thresholds were used to define the input to MEMERIS and * indicates that the more stringent threshold was selected. The last three columns contain the selected free parameter settings for MatrixREDUCE.
(0.01 MB PDF)

Table S3 Result of bootstrap analysis of relative AUC-PRs. Each entry represents the number of times RNAcontext has a larger/smaller AUC-PR value compared to AUC-PR values of MatrixREDUCE & MEMERIS on 1,000 bootstrap samples from the test set results (shown in Table 1). * indicates that the difference is not significant according to Wilcoxon's sign rank test.
(0.01 MB PDF)
