# Supplementary material for: RNAcontext: A New Method for Learning the Sequence and Structure Binding Preferences of RNA-Binding Proteins
Source: PLoS Comput Biol. 2010 Jul 1;6(7):e1000832. doi: 10.1371/journal.pcbi.1000832 (PMC2895634; doi:10.1371/journal.pcbi.1000832)
Supplement: Protocol S1 — A brief review of DNA motif finding. (0.16 MB PDF) [file pcbi.1000832.s001.pdf]

Protocol S1: A brief review of DNA motif finding

Hilal Kazan[1], Debashish Ray[2], Esther T Chan[3], Timothy R Hughes[2,3,4], Quaid Morris[1,2,3,4,*]

1 Department of Computer Science, University of Toronto, Toronto, Ontario, Canada

2 Banting and Best Department of Medical Research, University of Toronto, Toronto, Ontario, Canada

3 Department of Molecular Genetics, University of Toronto, Toronto, Ontario, Canada

4 Donnelley Centre for Cellular and Biomolecular Research, University of Toronto, Toronto, Ontario, Canada

$\ast$ **E-mail: quaid.morris@utoronto.ca**

In this protocol, we briefly review some aspects of DNA- and RNA-motif finding in order to help situate our new model within its broader theoretical context.

There are two major classes of motif model representations for sequence preferences of nucleic acid-binding (i.e. DNA-binding or RNA-binding) proteins (NBPs). The models in the first class (hereafter "word frequency (motif) models") represent a probability distribution over nucleic acid (NA) words. Models in the second class (hereafter "affinity-based (motif) models") parameterize a function that assigns the relative binding affinity (or log binding affinity) of the protein for NA words.

Typically, algorithms that learn word frequency models take as input a set of sequences enriched for NBPs binding sites and fit their models using maximum likelihood or penalized maximum likelihood methods. Though recently methods that use different point estimates of the parameter posterior have been developed [1, 2]. In contrast, most algorithms that learn affinity-based models directly also take as input a numerical value associated with each sequence. This value is interpreted as a measurement of the binding affinity of the NBP for the sequence. Typically, these types of models require examples of both bound and unbound sequences. These types of affinity data have become increasingly common [3–5] as have algorithms (including RNAcontext) to fit motifs using these values [6–11]. However, it is also possible to transform a word frequency model into an affinity-based model, as we describe below.

Word frequency motif models

A key step in fitting word frequency motif models is using the input sequences to generate counts of words that represent NBP binding sites; motif models are then fit to these counts. Often generating these word

counts, or expected word counts, is done as part of procedure that iterates between attempting to locate the NBP binding sites within the input sequences and refining the fit of the motif model [2,12].

Formally, let $\{[\text{P-}s_1], [\text{P-}s_2], \ldots, [\text{P-}s_N]\}$ be the counts (or expected counts) of how often various words $\{s_1, s_2, \ldots, s_N\}$ appear as binding sites of protein P in the input set. Let $Pr(s;\Theta)$ be a word frequency motif model where $Pr(s;\Theta)$ is a probability distribution over words s parameterized by Θ. Often, but not always, the support of $Pr(s;\Theta)$ is NA words of a fixed, pre-defined length K. The parameters Θ can be learned by optimizing the fit to the empirical word probabilities, i.e.,

$$Pr(s_i;\Theta) \approx \frac{[\text{P-}s_i]}{\sum_{j=1}^{N}[\text{P-}s_j]}, \forall i. \tag{1}$$

Until recently, NBP binding preferences were estimated from a relatively small number of binding sites defined *in vivo* or through low-throughput *in vitro* selection procedures. This small number of observations was insufficient to reliably estimate $Pr(s_i;\Theta)$ for each word, and as such word probabilities for fixed-length motif models were estimated using a product-multinomial model, commonly known as a position frequency matrix (PFM), in which the distributions of bases at each position in the word are independent. In the PFM, $Pr(s_i;\Theta) = \prod_{k=1}^{K} \Theta_{k,s_i(k)}$ where $s_i(k)$ indicates the k-th base in word s_i. This model only contains a small number of parameters, $3K$, and its maximum likelihood estimate is easily found by setting $\Theta_{k,s_i(k)} = f_{k,s_i(k)}$ where $f_{k,s_i(k)}$ is the frequency of $s_i(k)$ at position k. However, because PFM models are inaccurate representations of transcription factor (TF) binding affinity [13,14], a variety of more complex probability distributions have been developed to model interactions between bases [15–19] or variable-spacing between binding sites of obligatory heterodimers like bZIP or bHLH proteins [20].

Affinity-based motif models

These models are based on physical principles of protein-ligand interactions. In particular, consider the equilibrium reaction of binding of a protein P to the NA word s:

$$\text{P} + \text{s} \underset{k_{off}}{\overset{k_{on}}{\rightleftharpoons}} \text{P-}s \tag{2}$$

where k_{on} and k_{off} represents the protein binding and dissociation rates respectively. The binding affinity of the protein for s can be expressed in terms of its equilibrium constant $K_a(s)$:

$$K_a(s) = \frac{[\text{P-}s]}{[\text{P}][s]} = \frac{k_{on}}{k_{off}} \tag{3}$$

where [P], [s], [P-s] correspond to the concentrations of the unbound protein, unbound s, and the protein in complex with s respectively. Note that we are using the same notation for concentration as we do for word counts because these two quantities differ only in their units. Affinity-based motif finding methods fit parameters Ω of their motif models $W(s;\Omega)$ by trying to match their affinity estimates for a given word s those implied by the input, i.e. $W(s;\Omega) \approx K_a(s)$.

Note that, in addition to $K_a(s)$, motif models in this class have also been designed to estimate a number of other measures of binding affinity, e.g. the dissociation constant $K_d(s) = K_a(s)^{-1}$, the log binding affinity $\log K_a(s)$, or the relative binding affinity $CK_a(s)$ up to an unknown constant C that is independent of s [8]. The popular position weight matrix (PWM) [21, 22] or the position-specific affinity matrix (PSAM) [23] are examples of these types of models. Note also, that one can derive an estimate of relative binding affinity $W(s_i;\Theta)$ implied by a word frequency model, $Pr(s_i;\Theta)$ by dividing the latter by $[s_i]$, i.e.

$$W(s_i;\Theta) = \frac{Pr(s_i;\Theta)}{[s_i]} \approx \frac{\left([P\text{-}s_i] \Big/ \sum_{j=1}^{N} [\text{P-}s_j]\right)}{[s_i]} \propto K_a(s_i)$$

For example, MotifRegressor [6] uses this approach, approximating $[s_i]$ with a third-order Markov model trained on background sequence, to translate word frequency models fit by MDScan [24] into affinity-based models suitable for scoring sequences.

Estimating sequence affinity from word affinity

Rarely do the sequences input into motif finding algorithms consist of delineated binding sites. Furthermore, the input sequences ”enriched” for binding sites can contain more than one binding site, or possibly none at all. As such, an important component of any motif finding procedure, is a sequence scoring function that takes as input the probabilities or affinities assigned to each word by the motif model and outputs a ”score” for the entire sequence that reflects the number of likely NBP binding sites therein and their strength.

Word frequency motif models are usually paired with probabilistic generative models of sequences. The "score" computed by these generative models for an arbitrary sequence is the probability of generating the sequence under the model. These procedures are often subject to certain constraints about how the sequences were generated, such as the presence of exactly one, zero or one, or an arbitrary number of binding sites per sequence. In the MEME (and MEMERIS [25]) algorithm, for example, these three possibilities are called the OOPS, ZOOPS, and TCM options, respectively. Further refinements to generative models of sequences employ, for example, Hidden Markov Models to model steric hindrances that prevent overlapping binding sites and/or to model clustering of binding sites within cis-regulatory modules. A good summary of recent work in this area is provided in [26]. One advantage to this approach is that it is easy to incorporate competition for NBP binding sites from, for example nucleosomes [27] or internal RNA secondary structure [25], by assessing a prior probability on possible NBP binding sites according to the strength of competition for the site. A disadvantage to this approach is that the physical interpretation of these generative probabilities becomes difficult when there are multiple binding sites within a sequence.

There remains some controversy about the best approach for scoring sequences using affinity-based motif models. Early algorithms (e.g. [6–8]) used the sum of the affinities of each word in the sequence as an estimate of the NBP affinity for the entire sequence. One criticism of this approach is that the number of proteins bound to the sequence (also called the "occupancy" of the sequence) also depends on the number of proteins initially available for binding. So, if the initial protein concentration is low and the sequence has many high affinity binding sites, the actual occupancy of the sequence can be much lower than its potential occupancy implied by the estimated affinity. To address these concerns, some sequence scoring functions use affinity-based motif models to compute a function $N(s)$, the "occupancy" of word s [28–30], that also considers the initial concentration of proteins available for binding. This occupancy, which represents the proportion of words s bound by the protein, can be expressed as follows:

$$N(s) = \frac{[\text{P-}s]}{[\text{P-}s] + [s]}. \tag{4}$$

Note that dividing both sides with [P-s] gives:

$$N(s) = \frac{1}{1 + \dfrac{[s]}{[\text{P-}s]}} \tag{5}$$

Now we can rewrite the occupancy in terms of $K_d(S)$ since $\dfrac{[S]}{[\text{P-}s]}$ is equal to $\dfrac{K_d(S)}{[\text{P}]}$:

$$N(s) = \frac{1}{1 + \dfrac{K_d(s)}{[\text{P}]}} \tag{6}$$

which is equal to:

$$N(s) = \frac{1}{1 + \exp(\log K_d(s)) - \log[\text{P}])} \tag{7}$$

So, given an affinity-based motif model $W(s;\Omega)$ of binding affinity $K_a(s) = K_d(s)^{-1}$, measured under the same conditions, one can calculate an estimate $\hat{N}(s)$ of occupancy as follows:

$$\hat{N}(s) = \frac{1}{1 + \exp(-\log W(s;\Omega) - \log[\text{P}])}. \tag{8}$$

When fitting an affinity-based model using occupancy-based scoring, one can represent the often unknown constant log[P] with a bias β and train it while fitting the model. Note also, that one can adapt an existing affinity-based motif model $\tilde{W}$ for an NBP to predict occupancy under different experimental conditions (i.e. a change in temperature) by also introducing a scale α, so that the final model becomes:

$$\hat{N}(s) = \frac{1}{1 + \exp(-\alpha \log \tilde{W}(s;\Omega) - \beta)}. \tag{9}$$

In RNAcontext, we replace $\alpha \log \tilde{W}(s;\Omega) \approx \log K_d(S)$ with $\sum_{k=1}^{K} \Theta_{k,s(k)}$ where Θ represents the model parameters. So that we write equation 9 as follows:

$$N^{\text{seq}}(s) = \sigma(\sum_{k=1}^{K} \Theta_{k,s(k)} + \text{bias}) \tag{10}$$

where $\sigma(x) = 1/(1 + e^{-x})$ is the logistic function.

Occupancy-based sequence scoring functions include those that simply sum the occupancy of all words

in the sequence [30], those that calculate the probability that at least one site in the sequence is bound using the "noisy-OR" function [29], and more complex schemes that consider competitive and cooperative binding [29, 31].

References

1. Newberg LA, Thompson WA, Conlan S, Smith TM, McCue LA et al. (2007) A phylogenetic Gibbs sampler that yields centroid solutions for cis-regulatory site prediction. Bioinformatics 23(14): 1718-1727

2. Carvalho LE, Lawrence CE (2008) Centroid estimation in discrete high-dimensional spaces with applications in biology. Proc Natl Acad Sci USA, 105(9): 3209-3214

3. Ray D, Kazan H, Chan ET, Castillo LP, Chaudhry S et al. (2009) Rapid and systematic analysis of the RNA recognition specificities of RNA-binding proteins. Nat Biotech 27(7): 667-670.

4. Berger MF, Bulyk ML (2006) Protein binding microarrays (PBMs) for the rapid, high-throughput characterization of the sequence specificities of DNA binding proteins Methods Mol Biol 338: 245260.

5. Maerkl SJ, Quake SR (2007) A systems approach to measuring the binding energy landscapes of transcription factors. Science 315(5809): 233-237

6. Conlon EM, Liu XS, Lieb JD, Liu JS (2002) Integrating regulatory motif discovery and genome-wide expression analysis Proc Natl Acad Sci U S A 100(6): 3339-3344

7. Bussemaker H.J., Li,H. and Siggia, ED (2001) Regulatory element detection using correlation with expression. Nature Genet 27: 167171.

8. Foat BC, Morozov AV, Bussemaker HJ (2006) Statistical mechanical modeling of genome-wide transcription factor occupancy data by MatrixREDUCE. Bioinformatics 22: e141e149.

9. Chen X, Hughes TR, Morris Q (2006) RankMotif++: a motif search algorithm that accounts for relative ranks of k-mers in binding transcription factors. Bionformatics 23: 72-79.

10. Tanay, A (2006) Extensive low-affinity transcriptional interactions in the yeast genome. Genome Res. 16: 962972

11. Eden E, Lipson D, Yagev S, Yakhini Z (2007) Discovering motifs in ranked lists of DNA sequences. PLoS Comput Biol 3:e39

12. Bailey TL, Williams N, Misleh C, Li WW (2006) MEME: discovering and analyzing DNAand protein sequence motifs. Nucleic Acids Res 34: W369373.

13. Benos P, Bulyk ML, Stormo GD (2002). Additivity in protein-DNA interactions: how good an approximation is it? Nucleic Acids Res 30(20):4442-4451.

14. Bulyk ML, Johnson PLF, Church GM (2002) Nucleotides of transcription factor binding sites exert interdependent effects on the binding affinities of transcription factors. Nucleic Acids Res 30(5):1255-1261.

15. Barash Y, Elidan G, Friedman N, Kaplan T (2003) Modeling dependencies in proteinDNA binding sites. In: Proceedings of the Seventh Annual International Conference on Research in Computational Molecular Biology. New York: ACM Press.. pp 2837.

16. King OD, Roth FP(2003) A non-parametric model for transcription factor binding sites. Nucleic Acids Res 31(19):e116.

17. Sharon E, Lubliner S, Segal E (2008) A feature-based approach to modeling protein-DNA interactions PLoS Comp Biol 4(8): e1000154

18. Grundy WN, Bailey TL, Elkan CP, Baker ME (1997) Meta-MEME: motif-based hidden markov models of biological sequences. Computer Applications in the Biosciences, 13(4): 397-406

19. Eddy SR (1998) Profile hidden Markov models. Bioinformatics 14(9): 755-763

20. Liu X, Brutlag DL, Liu JS (2001) BioProspector: discovering conserved DNA motifs in upstream regulatory regions of co-expressed genes. Pac Symp Biocomput:127-138

21. Berg OG, von Hippel PH (1987) Selection of DNA binding sites by regulatory proteins. Statistical-mechanical theory and application to operators and promoters. J Mol Biol 193: 723-750

22. Stormo GD (2000) DNA binding sites: representation and discovery. Bioinformatics 16(1): 16-23

23. Foat BC, Houshmandi SS, Olivas WM, Bussemaker HJ (2005) Profiling condition-specific, genome-wide regulation of mRNA stability in yeast. Proc Nat Sci Acad USA 102(49): 17675-17680

24. Liu XS, Brutlag DL, Liu JS (2002) An algorithm for finding protein-DNA binding sites with applications to chromatin immunoprecipitation microarray experiments. Nat Biotechnol 20(8): 835-9.

25. Hiller M, Pudimat R, Busch A, Backofen R (2006) Using RNA secondary structures to guide sequence motif finding towards single-stranded regions. Nucleic Acids Res 34(17): e117

26. Tang MH, Krogh A, Winther O (2008) BayesMD: flexible biological modeling for motif discovery. J Comput Biol 15(10):1347-63.

27. Narlikar L, Gordân R, Hartemink AJ (2007) Nucleosome occupancy information improves de novo motif discovery. In: Proceedings of the Eleventh Annual International Conference on Research in Computational Molecular Biology. New York: ACM Press..107-121

28. Segal E, Barash Y, Simon I, Friedman N, Koller D (2002) From promoter sequence to expression: a probabilistic framework. In: Proceedings of the Sixth Annual International Conference on Research in Computational Molecular Biology. New York: ACM Press.. 263-272.

29. Granek JA, Clarke ND (2005) Explicit equilibrium modeling of transcription-factor binding and gene regulation. Genome Biology 6: R87.

30. Roider HG, Kanhere A, Manke T, Vingron M (2007) Predicting transcription factor affinities to DNA from a biophysical model. Bioinformatics 23(2):134-141.

31. Segal E, Raveh-Sadka T, Schroeder M, Unnerstall U, Gaul U (2008) Predicting expression patterns from regulatory sequence in Drosophila segmentation. Nature 451: 535-540
