# Supplementary material for: RNAcontext: A New Method for Learning the Sequence and Structure Binding Preferences of RNA-Binding Proteins
Source: PLoS Comput Biol. 2010 Jul 1;6(7):e1000832. doi: 10.1371/journal.pcbi.1000832 (PMC2895634; doi:10.1371/journal.pcbi.1000832)
Supplement: Table S1 — Properties of the sequences in the input sets. The composition of Sets A and B in terms of relative proportions of stem-loops and weakly structured sequences among their Positive, Negative and Other groups. The input sets are partitioned into these three groups according to their RNAcompete-measured affinities. The sequences with affinities above a threshold are defined as Positive; the sequences with affinities below the median affinities over all the sequences in the given set are defined as Negative and the remaining sequences are placed in the Other group. Within each group, the number of weakly structured sequences and stem-loops are displayed. For RNAcontext and MatrixREDUCE all the sequences in Positive, Negative and Other categories are used for training whereas when running MEMERIS, only Positive sequences are used for training. The test sets are comprised of all sequences in the Positive and Negative groups. (0.01 MB PDF) [file pcbi.1000832.s004.pdf]

Proteins	Set A						Set B					
	positive		negative		other		positive		negative		other	
	weak	stem-loop	weak	stem-loop	weak	stem-loop	weak	stem-loop	weak	stem-loop	weak	stem-loop
Vts1p	289	260	16,863	3409	11,390	9,107	282	239	16,697	3373	11,136	8,885
SLM2	520	1	18,373	9,984	19,109	14,819	566	5	18,143	10,100	18,879	14,671
YB1	555	59	15,985	11,838	29,317	21,858	519	62	15,701	11,784	29,177	21,905
RBM4	726	6	16,762	9,798	17,140	9,231	792	14	16,655	9,659	16,841	9,127
SF2	523	71	17,961	12,616	22,713	11,922	529	92	17,867	12,282	22,378	12,017
FUSIP1	201	436	21,181	4,148	9,918	15,149	234	426	20,986	4,096	9,928	15,185
HuR	608	1	16,379	13,422	24,877	4,348	586	0	15,944	13,332	25,095	4,405
U1A	310	322	8,232	325	6,752	1,740	277	328	8,250	337	6,665	1738
PTB	467	62	21,315	6,908	18,040	15,356	448	72	21,309	6,820	17,779	15,332
