# Supplementary material for: RNAcontext: A New Method for Learning the Sequence and Structure Binding Preferences of RNA-Binding Proteins
Source: PLoS Comput Biol. 2010 Jul 1;6(7):e1000832. doi: 10.1371/journal.pcbi.1000832 (PMC2895634; doi:10.1371/journal.pcbi.1000832)
Supplement: Table S2 — Details about the chosen models for RNAcontext, MEMERIS and MatrixREDUCE. Optimal free parameter settings for RNAcontext, MEMERIS and MatrixREDUCE. The column Set describes the training set and contains either weak or full where weak indicates that motifs were trained on the weakly structured sequences and full indicates that motifs were trained on the full set of sequences. The columns, MW-A and MW-B, show the selected motif length for the test sets A and B respectively. There is an extra other column for MEMERIS which shows the other free parameters that are chosen. Namely, EF and PU are two different ways to measure single-strandedness of a region; OOPS (exactly one motif occurrence per sequence), ZOOPS (zero or one motif occurrence per sequence), and TCM (zero or more motif occurrence per sequence) are options (-mod) that indicate the expected number of motifs per sequence. The values in the next column (i.e. 0.1 or 1) are the chosen pseudocount parameters among the available values 0.1, 1, 3. The lower the pseudocount value, the more impact the single-strandedness of the binding site has in the model. Two different thresholds were used to define the input to MEMERIS and * indicates that the more stringent threshold was selected. The last three columns contain the selected free parameter settings for MatrixREDUCE. (0.01 MB PDF) [file pcbi.1000832.s005.pdf]

| Proteins | RNAcontext | | | MEMERIS | | | | | MatrixREDUCE | | |
|---|---|---|---|---|---|---|---|---|---|---|---|
| | Set | MW-A | MW-B | Set | MW-A | Other-A | MW-B | Other-B | Set | MW-A | MW-B |
| Vts1p | full | 7 | 7 | full | 7 | EF ZOOPS 0.1 * | 7 | EF ZOOPS 0.1 * | full | 8 | 9 |
| SLM2 | weak | 8 | 8 | full | 11 | PU ZOOPS 0.1 | 5 | PU OOPS 3 | weak | 8 | 9 |
| YB1 | full | 8 | 8 | weak | 8 | EF ZOOPS 1 | 12 | EF ZOOPS 1 | full | 8 | 8 |
| RBM4 | full | 6 | 8 | weak | 4 | EF OOPS 3 | 4 | PU ZOOPS 3 | full | 7 | 7 |
| SF2 | weak | 5 | 5 | weak | 4 | PU OOPS 1 * | 4 | EF OOPS 3 | full | 4 | 5 |
| FUSIP1 | full | 12 | 11 | full | 6 | EF TCM 0.1 * | 6 | EF TCM 0.1 | full | 8 | 8 |
| HuR | weak | 9 | 9 | full | 10 | PU OOPS 0.1 | 12 | PU OOPS 1 | full | 8 | 8 |
| U1A | weak | 9 | 9 | weak | 12 | EF ZOOPS 0.1 | 8 | EF ZOOPS 0.1 * | full | 8 | 9 |
| PTB | weak | 7 | 7 | weak | 12 | EF OOPS 3 | 12 | EF OOPS 1 | weak | 7 | 5 |
