# Supplementary material for: RNAcontext: A New Method for Learning the Sequence and Structure Binding Preferences of RNA-Binding Proteins
Source: PLoS Comput Biol. 2010 Jul 1;6(7):e1000832. doi: 10.1371/journal.pcbi.1000832 (PMC2895634; doi:10.1371/journal.pcbi.1000832)
Supplement: Table S3 — Result of bootstrap analysis of relative AUC-PRs. Each entry represents the number of times RNAcontext has a larger/smaller AUC-PR value compared to AUC-PR values of MatrixREDUCE & MEMERIS on 1,000 bootstrap samples from the test set results (shown in Table 1). * indicates that the difference is not significant according to Wilcoxon's sign rank test. (0.01 MB PDF) [file pcbi.1000832.s006.pdf]

Proteins	MatrixREDUCE		MEMERIS	
	A	B	A	B
RBM4	1000/0	1000/0	1000/0	1000/0
FUSIP1	1000/0	1000/0	1000/0	1000/0
Vts1p	990/10	999/1	996/4	959/41
YB1	1000/0	995/5	1000/0	1000/0
SLM2	974/26	803/197	1000/0	1000/0
SF2	983/117	875/125	1000/0	1000/0
U1A	1000/0	504/496*	999/1	295/705
HuR	978/22	996/4	1000/0	1000/0
PTB	213/787	961/39	1000/0	1000/0
